# Assessing Lymphatic Filariasis Data Quality in Endemic Communities in Ghana, Using the Neglected Tropical Diseases Data Quality Assessment Tool for Preventive Chemotherapy

**DOI:** 10.1371/journal.pntd.0004590

**Published:** 2016-03-30

**Authors:** Dziedzom K. de Souza, Eric Yirenkyi, Joseph Otchere, Nana-Kwadwo Biritwum, Donne K. Ameme, Samuel Sackey, Collins Ahorlu, Michael D. Wilson

**Affiliations:** 1 Parasitology Department, Noguchi Memorial Institute for Medical Research, University of Ghana, Accra, Ghana; 2 Ghana Field Epidemiology and Laboratory Training Programme, School of Public Health, University of Ghana, Accra, Ghana; 3 National NTDs Control Program, Ghana Health Service, Accra, Ghana; 4 Epidemiology Department, Noguchi Memorial Institute for Medical Research, University of Ghana, Accra, Ghana; Centers for Disease Control and Prevention, UNITED STATES

## Abstract

**Background:**

The activities of the Global Programme for the Elimination of Lymphatic Filariasis have been in operation since the year 2000, with Mass Drug Administration (MDA) undertaken yearly in disease endemic communities. Information collected during MDA–such as population demographics, age, sex, drugs used and remaining, and therapeutic and geographic coverage–can be used to assess the quality of the data reported. To assist country programmes in evaluating the information reported, the WHO, in collaboration with NTD partners, including ENVISION/RTI, developed an NTD Data Quality Assessment (DQA) tool, for use by programmes. This study was undertaken to evaluate the tool and assess the quality of data reported in some endemic communities in Ghana.

**Methods:**

A cross sectional study, involving review of data registers and interview of drug distributors, disease control officers, and health information officers using the NTD DQA tool, was carried out in selected communities in three LF endemic Districts in Ghana. Data registers for service delivery points were obtained from District health office for assessment. The assessment verified reported results in comparison with recounted values for five indicators: number of tablets received, number of tablets used, number of tablets remaining, MDA coverage, and population treated. Furthermore, drug distributors, disease control officers, and health information officers (at the first data aggregation level), were interviewed, using the DQA tool, to determine the performance of the functional areas of the data management system.

**Findings:**

The results showed that over 60% of the data reported were inaccurate, and exposed the challenges and limitations of the data management system. The DQA tool is a very useful monitoring and evaluation (M&E) tool that can be used to elucidate and address data quality issues in various NTD control programmes.

## Author Summary

The Global Programme for the Elimination of Lymphatic Filariasis has been conducting yearly treatment of entire communities in endemic countries since the year 2000. During the treatments various information is collected on the populations, number of medicine tablets distributed and remaining, the number of people treated, etc. that can be used to evaluate the performance of the lymphatic filariasis control programme. For example, information on the number of people treated in a District gives an indication of the success of the programme. In line with this, the World Health Organization in collaboration with other agencies developed a tool for Neglected Tropical Diseases (NTD) to help national control programmes assemble and analyse their data. This study was undertaken to evaluate this tool and the information collected from some endemic communities in Ghana. Community registers were reviewed and personnel involved in drug distribution in the communities were interviewed to collect the necessary information. The results showed that more than half of the data reported in the endemic communities surveyed were inaccurate. It also revealed some weaknesses in the data management and reporting system. The tool, however, is good for identifying and quantifying the magnitude of the challenges encountered in the information management for NTD programmes, especially at peripheral levels.

## Introduction

The Global Programme to Eliminate Lymphatic Filariasis (GPELF) started its activities in the year 2000, with the aims of eliminating lymphatic filariasis (LF) as a public health problem by the year 2020, through mass drug administration (MDA) in endemic implementation units (IU) [1]. In many countries significant progress has been made in controlling the disease; however, many programmatic challenges continue to affect the performance of National LF Control Programmes. Notable among these is the effective implementation of the preventive chemotherapy strategy in endemic communities [2, 3].

The quality of data reported in healthcare systems is important for evaluating programmes, as such high quality health information is crucial in addressing global health challenges and building strong public health systems [4]. Data Quality Assessment (DQA) is a scientific and statistical evaluation of data to determine if they meet the objectives, and are of the right type, quality, and quantity to support their intended use [5]. At present yearly MDA has been undertaken in 53 of the 73 LF endemic countries [6]. During the MDA various data are collected at various levels to help in the planning and improvement of activities. As such high quality becomes the prerequisite for better information, better decision-making and better population health [7].

In all LF endemic Districts in Ghana, various information are collected during MDAs, including number of treatments given, number of people treated, number of tablets used, reasons for non-treatment, place of treatment, individual identification (name and address), name of drug used, age and sex of drug recipients, etc. Public health data can be useful for decision-making, effective service delivery, and evaluating prevailing programmes in order to maintain high quality of healthcare. Poor data quality not only contributes to poor decisions and loss of confidence in the systems, but also affects the validity of impact evaluation studies [8]. Furthermore, variability in data quality from health management information systems in sub-Saharan Africa threatens utility of these data as a tool to improve health systems [9]. Thus, collecting accurate data will aid appropriate intervention for elimination.

The WHO, in collaboration with NTD partners, including ENVISION/RTI, developed a DQA tool to identify and characterize challenges with NTD data quality–including incomplete and inaccurate data or data not timely reported–following a recommendation from the WHO Working Group on Monitoring and Evaluation of Preventive Chemotherapy. The DQA tool focuses exclusively on verifying the quality of reported data quantitatively and assessing the underlying data management and reporting systems qualitatively for standard programme-level output indicators. Data quality dimensions include accuracy, reliability, completeness, timeliness, precision, integrity and confidentiality [10]. In 2014, training was undertaken to introduce the tool to various stakeholders, with field testing to follow [10]. This study was undertaken to evaluate the quality of data reported in selected communities or service delivery points (SDP), and the data management functions and capabilities in three LF endemic Districts in Ghana.

## Methods

### Ethical Statement

Approval for this study was obtained from the Ethical Review Board of the Noguchi Memorial Institute for Medical Research (IRB 077/13-14). The District Health Office was informed of the study and permission sought to assess data from the registers. Written informed consent was obtained from all individuals interviewed during the study.

### Study Sites

**Fig 1:**
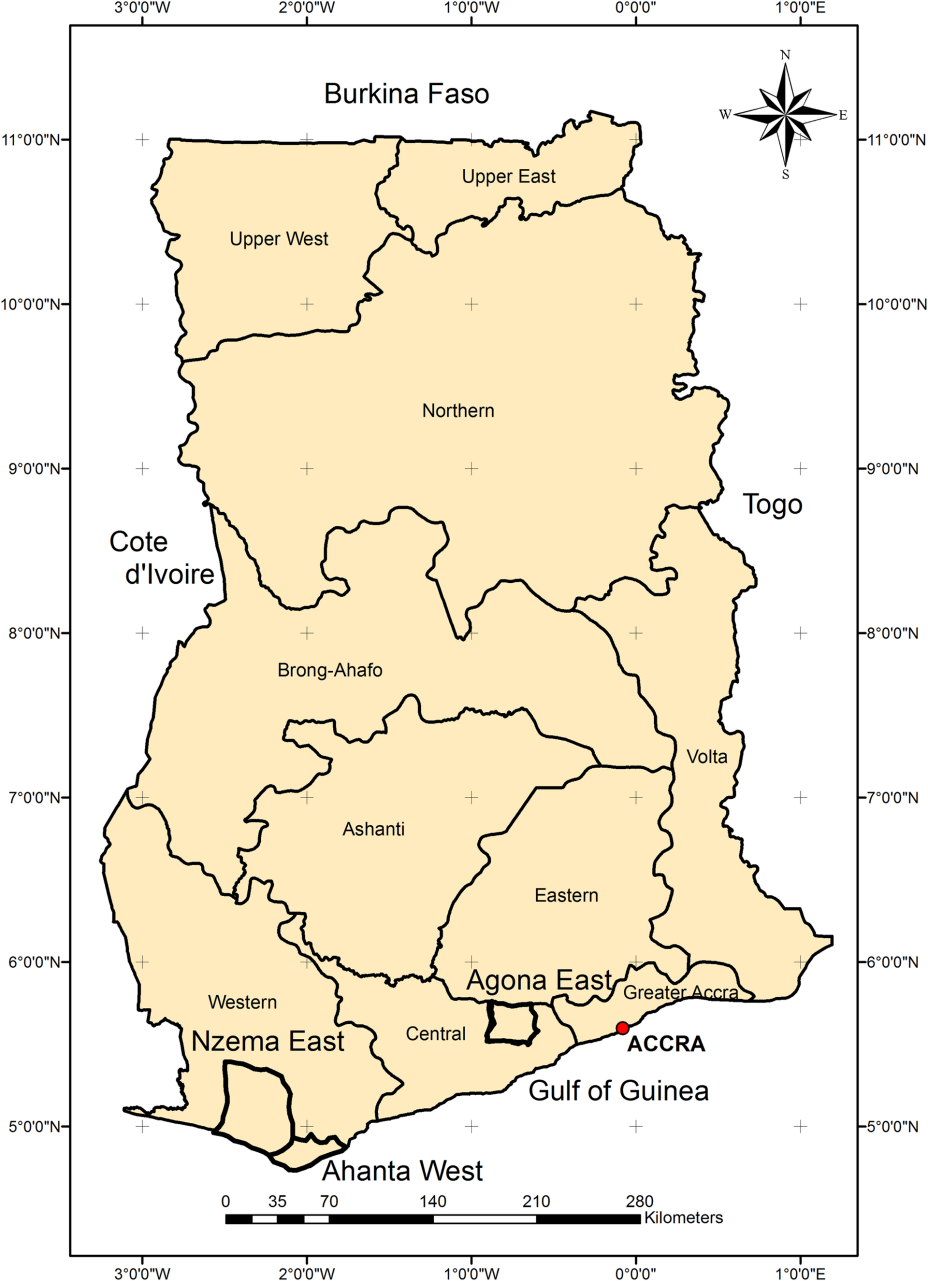
Map of Ghana showing the study districts.

This study was undertaken in the Ahanta West, Nzema East and Agona East Districts of Ghana (Fig 1). The Ahanta West and Nzema East Districts, both located in the Western Region of Ghana, started MDA in the years 2000 and 2002, respectively. Both districts represent areas with persistent transmission, with MDA ongoing at the time of this study in 2014. The Agona East District is located in the Central Region of Ghana and started MDA activities in 2002. By 2010, LF infection rate in Agona East District was considered to be below the 2% antigenemia and 1% microfilaremia thresholds required to stop MDA [11]. As such, treatment in Agona East ceased in 2010. Thus, for the purpose of comparing data between sites, the 2010 data registers were analysed for all the sites surveyed. While the DQA tool advocates the selection of sites based on probability proportionate to size (PPS), the survey communities in this study were randomly selected because the population estimates of the communities could not be obtained beforehand. Eight sites were surveyed in the Ahanta West District and 6 sites from the Nzema East and Agona East Districts respectively.

### Data Review and Interviews

A cross sectional study involving the review of data registers and interview of drug distributors, disease control officers and health information officers was done. Data registers at the SDPs capture data during MDA for compilation by health workers. Information contained in these registers includes age, sex, height, number of households, population, drug used, number of tablets used, number of tablets received, etc. While the tool is capable of being used at the SDPs and all intermediate data aggregation levels (IALs), in this study only the SDPs were evaluated for data quality since they represent the first data collection and handling locations. In assessing the data management systems and functions, the intermediate data aggregation level 1 (IAL-1) represents the last point for information dissemination into the SDPs, and the first point of data collection from the SDPs. The interview tool used is a standard questionnaire, developed as part of the DQA tool, with scoring guidelines coded 3 for “Yes, completely”, 2 for “Partly” and 1 for “No, not at all”. These scores take into consideration the response from all the interviewees. The DQA tool and further information can be obtained from the WHO Department of Control of Neglected Tropical Diseases.

To evaluate the quality of data reported in the study areas, data registers for SDPs for 2010 were obtained from IAL-1, for assessment. The assessment verified reported results (from IAL-1) in comparison with recounted values (from SDPs) for five indicators, i.e. number of tablets received, number of tablets used, number of tablets remaining, MDA coverage, and population treated. Sources of data for the five indicators were examined to determine the percentage of reports that were available, on-time, completed, collected and measured consistently, protected from deliberate bias, and maintained according to national or international standard. Further, drug distributors, disease control officers and health information officers available at the time of the study (at the first data aggregation level) and who were willing, were interviewed using the DQA tool, to determine the M&E structure, functions and capabilities, indicator definitions, links with national reporting system, data management processes and data collection and reporting forms and tools.

### Data Analysis and Interpretation

For each of the five indicators assessed a verification factor (VF) was calculated as the ratio of recounted value (from the data register) of the indicator to the reported value, expressed as a percentage. A value of 100% indicates a high level of accuracy. Values above 100% indicate under reporting, whiles values below 100 suggest over reporting. In interpreting the results, indicator values between 95–105% across multiple sites were considered as high quality reporting. Indicator values less than 90% and greater than 110% were considered poor quality reporting. Verification factors above 300 were excluded from the analysis. The values were compiled and graphs generated in GraphPad Prism version 6.05. Statistical significance was set at p-values less than 0.05.

Scores for the functional areas in the Data Management Assessment were automatically computed by the DQA tool, which also generates a spider chart. At individual sites, functional areas with scores >2.5 indicate high quality, whiles scores<2.0 reflect low quality. However, when comparing scores across sites, scores> = 2.8 indicate good performance and scores < = 1.5 indicate that a functional area needs to be improved.

## Results

In each District, 3–4 days were spent in reviewing and evaluating the data, and conducting interviews. The SDPs were visited by the study team to review the selected indicators from the community registers. It is worth mentioning that except for being interviewed by the study team, individuals working at any level with the LF control programme were not directly involved in the use of the tool, and the study was undertaken independently by personnel from the Noguchi Memorial Institute for Medical Research (NMIMR)–University of Ghana. Further, the evaluation of the tool took advantage of ongoing parasitological and entomological surveys in the Districts.

**Fig 2:**
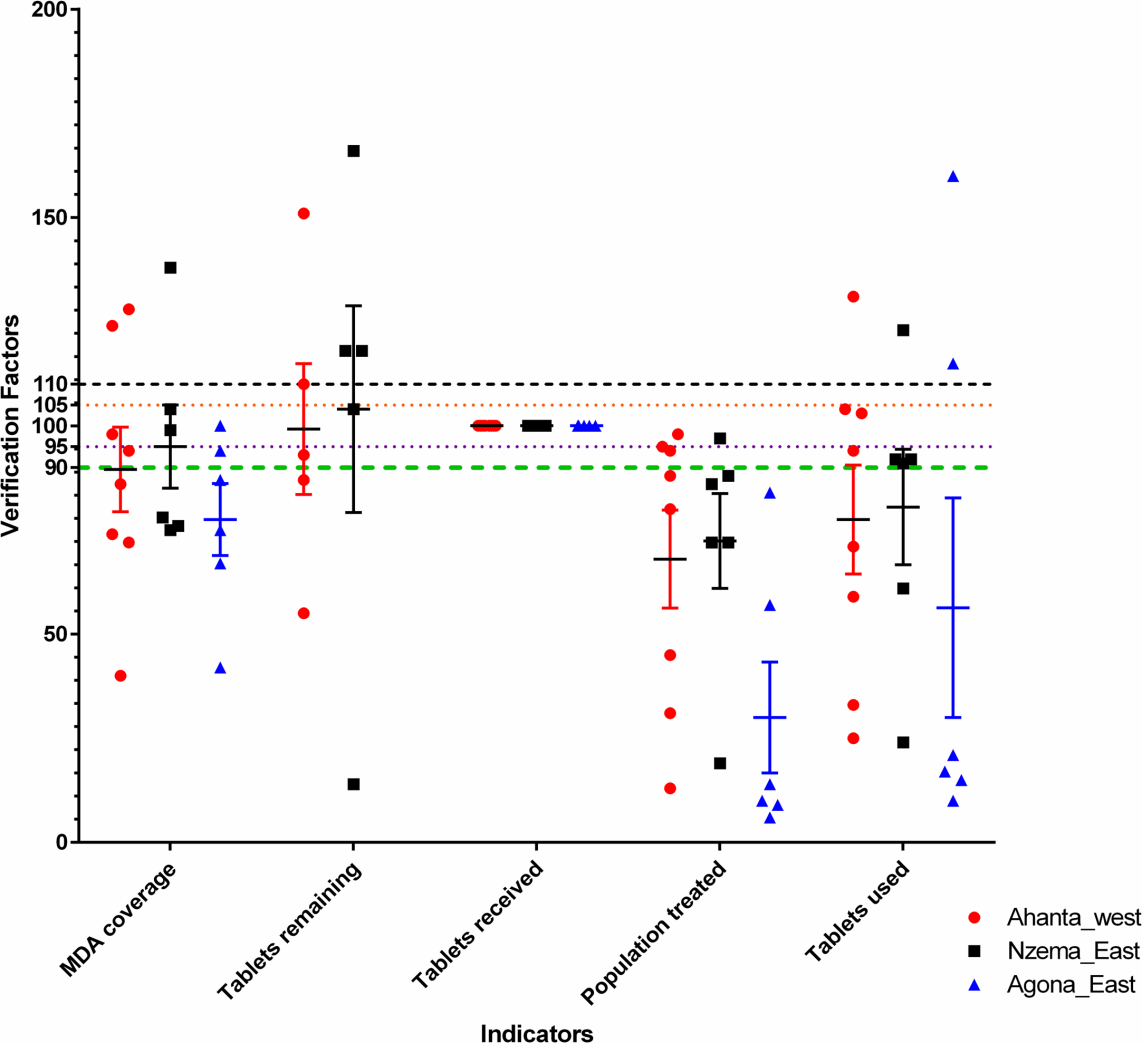
Verification factors for the indicators assessed in the various districts. Values between 95 and 105 are considered high quality, while values below 90 and above 110 are considered poor quality, with values <90 indicative of over reporting and values >110 indicative of under reporting. Points represent the values obtained for each community. For each District, the standard error bar is shown, with the short horizontal line across it representing the mean. 10 data points with verification factor above 300 were excluded from the analysis, and these include the Tablets remaining for Agona East.

The results showed that 40% (40/100) of all data examined were over reported while 22% (22/100) were under reported. The only consistent indicator that was accurately reported across sites was the number of tablets received. For the five indicators assessed, the VF were plotted for comparison between Districts (Fig 2). Between Districts, there was no significant difference in the indicators assessed, except for the population treated in Agona East.

**Fig 3:**
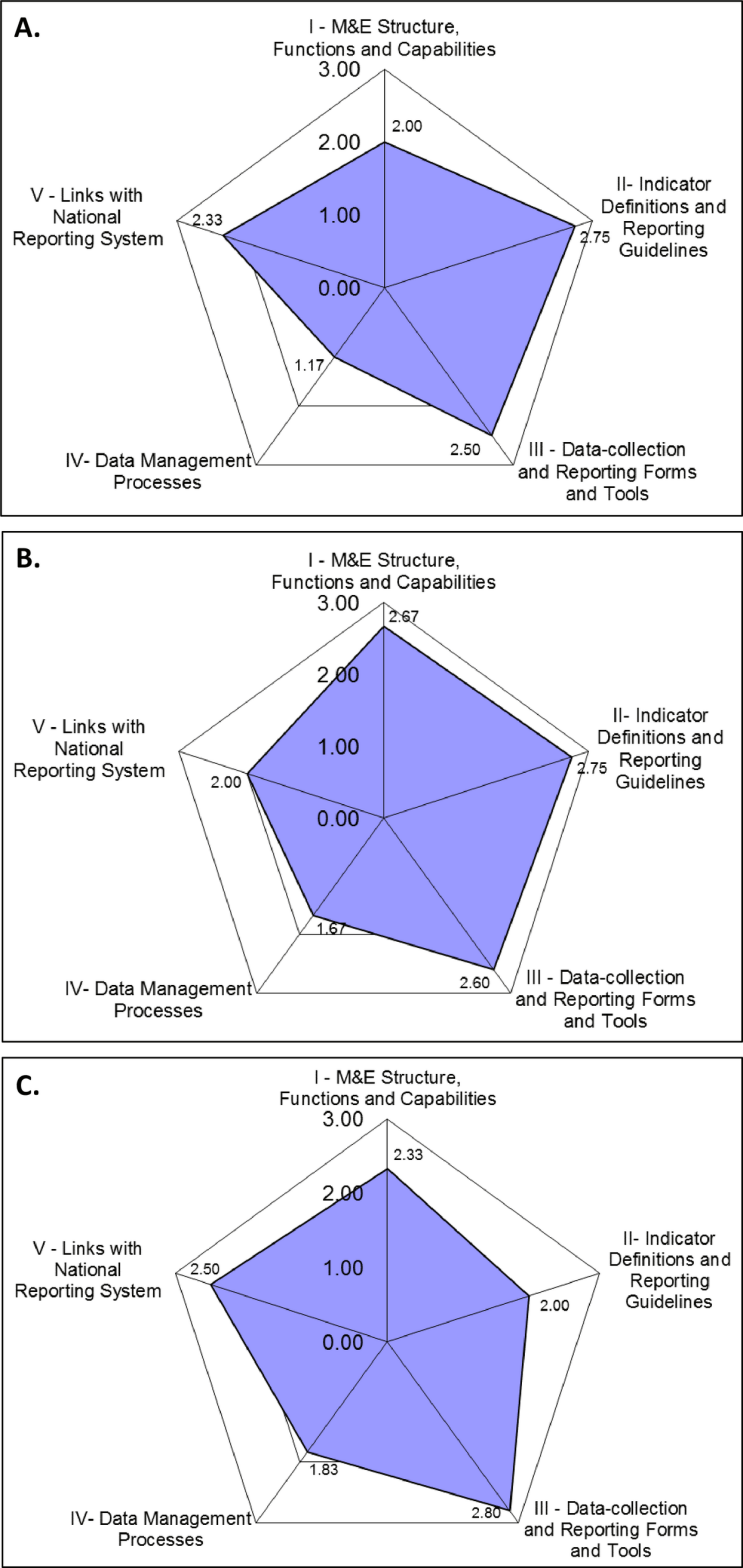
Functional areas of the data management assessment. A.) Agona Nkwanta DHMT, Ahanta West; B.) Axim DHMT, Nzema East; C.) Konyarko Health Post, Agona East. Functional areas with scores >2.5 indicate high quality, whiles score <2.0 reflect low quality. Scores ≥2.8 across sites, indicate good performance and scores ≤1.5 across sites indicates that functional area needs to be improved.

Results of the data management assessment in the Districts are shown in Fig 3. In Agona Nkwanta, indicator definitions and reporting guidelines were the strongest functional areas followed by data collection and reporting forms and tools. Data management processes was the weakest functional area, followed by M&E structure, function and capabilities. In Axim District Health Directorate, the strongest functional area was indicator definitions and reporting guidelines, followed by M&E structure, functions and capabilities then data-collection and reporting forms and tools. In Konyarko health post, the strongest functional area was the data collection and reporting forms and tools.

**Fig 4:**
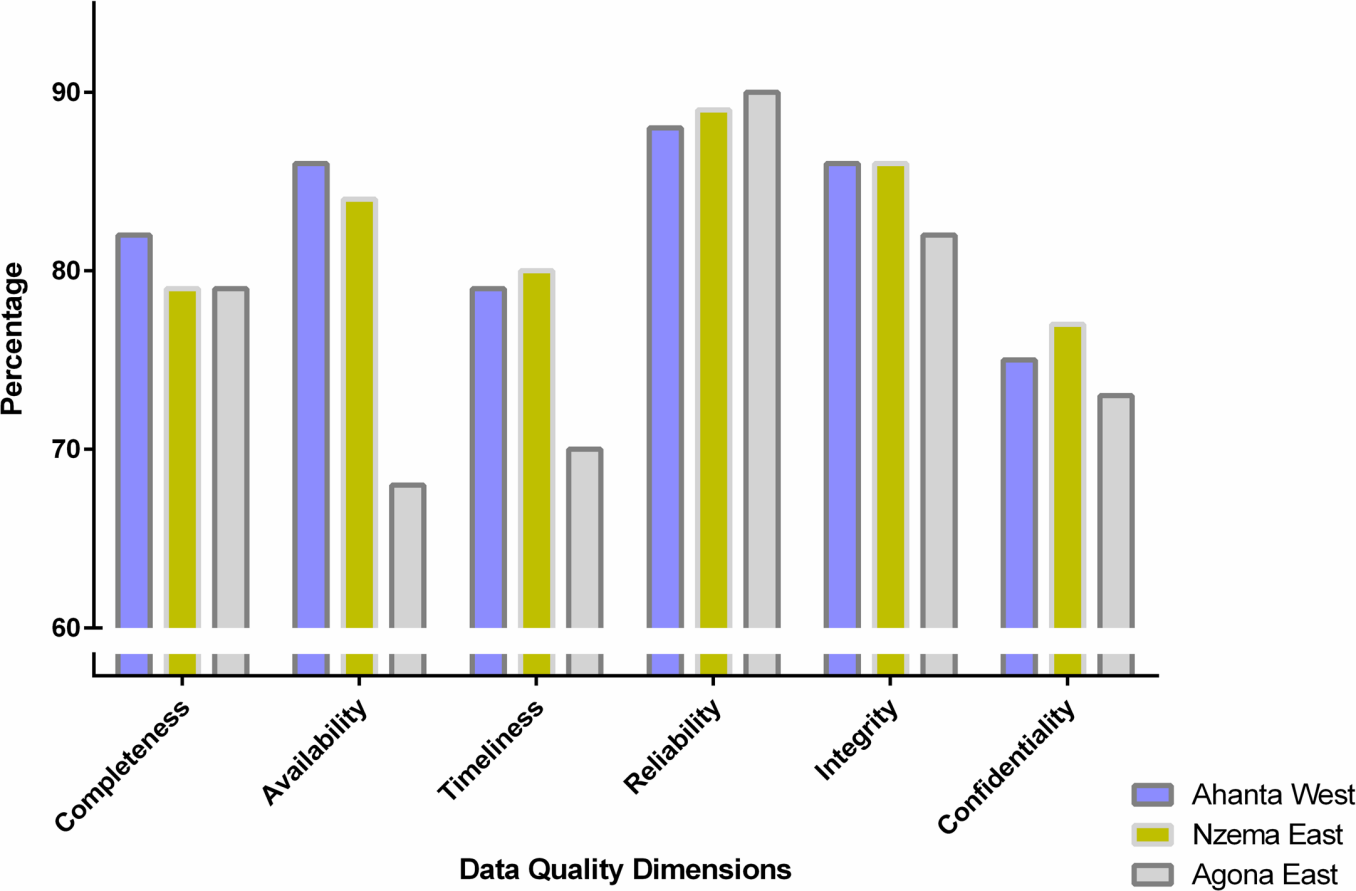
Data quality dimensions in study sites. Percentages indicate the score for each Data Quality Dimensions observed in the Districts.

In terms of the data quality dimensions, the observed values were more or less consistent between sites (Fig 4). In Ahanta West, the lowest reporting performance was confidentiality (75%), followed by timeliness (79%). However, the best reporting performance was reliability (88%), followed by availability (86%) and integrity (86%). In Nzema East District, the lowest reporting performance was confidentiality (77%), followed by completeness (79%). On the other hand, the best reporting performance was reliability (89%), followed by integrity (86%). In Agona East, the lowest reporting performance was availability (68%), followed by timeliness (70%), while the best reporting performance was reliability (90%), and followed by integrity (82%).

## Discussions

Data are vital to public health, since they signify and provide a documented account of public health practice. The extensive application of data, for the evaluation of public health responsibility and performance, highlights the importance of data quality and how to evaluate it. This study reflected the poor quality of data reported following MDA for LF, indicated as the over reporting or under reporting of the indicators assessed. In particular, is the overestimation of MDA coverage in many of the sites surveyed. Overestimation of MDA coverage in NTD programmes has been reported in many studies [12–14]. MDA coverage is the core indicator required for global reporting on preventive chemotherapy, thereby reflecting the performance of control programmes [14] and must therefore be strictly monitored. It is important to note that before MDA, drug distributors are gathered at the District level for training and orientation. In some of the communities, the drug distributors recalled having supervision support during the MDA, while this was not the case in other communities.

In terms of the functional areas of the LF control programme, even though study sites registered a score >2.0, which is considered passable, no site demonstrated a high quality data reporting system, given that no site scored >2.5 for all functional areas. However, in some of the surveyed areas, health workers reported that strict guidelines were received, defining the indicator to report on, where, when and to whom to send reports, and these guidelines have been vividly stated and followed. Additionally, this study found that community drug distributors always used a standard data capture tool, made available at the national level. t. Training plans, and trained data management staff were also available in some of the areas. On the other hand, data quality controls, and back-up procedures, confidentiality of personal data and feedback on data quality were not available. Moreover, trained data management staff and training plans were not sufficient, as well as non-availability of M&E organizational structure. Results from this study suggest that data management processes is the weakest functional area across sites. The reasons for this must further be investigated and addressed accordingly. Similar gaps, such as lack of M&E guidelines, poor feedback given to sub-national levels, poor data use and poor general data management capacity, lack of training programmes to build M&E skills, few standard practices related to confidentiality and document storage, have been reported following DQA in other countries [15, 16].

Overall, data confidentiality, completeness and timeliness require improvement in terms of reporting performance. In the survey sites, data was not managed according to protection and use standard and in most cases data were not reported on time. The completeness of data also needs to be improved as the data examined had missing reports. The data quality dimensions reported in this study are somewhat comparable to those reported elsewhere [17–19] and may indicate the general quality of data in health care systems. The validity of data reported also varied with the various indicators assessed. The most accurately reported indicator was number of tablets received. This is because the supply of drugs to communities was strictly supervised by the Districts.

With the renewed commitment from pharmaceutical companies and other NTD partners at the London Declaration on NTDs in 2012 [20], emphasis must be placed on value for money to ensure that the resources invested are worthwhile. The expanded drug donations and the programme goals presented in the NTD roadmap for implementation point to the importance of having a robust monitoring and reporting system, from the point of treatment by a drug distributor to the national and international levels [10, 21]. While some communities reported good quality data for some of the indicators assessed in this study, the majority of the indicators assessed were of poor quality, necessitating the need to get all communities up to standard. Thus, an important programmatic application of the DQA is to enable objective identification of context-specific issues that compromise data challenges and thereby trigger corrective action before the subsequent MDAs. For example, the use of the tool in LF may help inform how long to treat communities if measures are put in place to address challenges in order to consistently attain the required 65% MDA coverage rates, thus reducing the cost involved in undertaking further yearly treatment beyond the recommended 5–6 years, as per WHO guidelines [1, 22]. While Agona East appears to have the poorest data quality, it is the one District where MDA has been stopped. It is plausible that other factors such as baseline disease prevalence, vector competence and non-compliance to MDA may be at play [23–25], prompting the need for continued treatment in Districts with persistent transmission. As such, the link between data quality and programme success/failure needs to be further evaluated. Nonetheless, this tool may complement the Transmission Assessment Surveys (TAS) undertaken to inform on the need to stop MDA [26], by ensuring that the reported MDA coverage rates have truly been achieved in the evaluation units under assessment. Similarly, donated drugs must be properly accounted for, to ensure that they are put to their intended use, while monitoring the population treated may help in forecasting and budgeting for future MDAs.

While other assessment methods rely on the use of questionnaires and ability of the study respondents to recall past events [13, 14, 27], such as having taken the drug, this tool relies on examining and recounting data recorded in community registers. Though the former method may be subject to proper description of the specific MDA by the questionnaire administrator (considering the various treatment regimen for different NTDs) and the honesty of the study respondents, the DQA tool relies on data recorded for each individual in the register at the SDP. Thus, the tool may be considered as providing a more reliable estimate of indicators for assessing programme performance, with the ability to compare retrospective to current data. However, it is important to note that the tool is also limited to the raw data recorded, such that incorrect entries (especially individual records such as age and the number of tablets given to a particular person) in community registers cannot be detected using the tool.

In this study, PPS sampling wasn't used. Thus, the findings are only applicable to the areas under study, even though it is likely that the same findings occur nationwide. Nevertheless this would need to be confirmed by doing DQA with a representative sampling methodology. Further, in the Districts, data from 2010 was evaluated. While the evaluation of retrospective data can provide valuable information, the use of the tool for programme evaluation should consider the most up-to-date data in order for challenges to be resolved in real-time. In addition to these challenges in the study, the WHO protocol requires co-implementation with Ministry of Health (MoH), NTD programme and other NTD partners operating in the country. This study was undertaken as a research activity, with limited funding, taking advantage of on-going surveys in the study areas. As such, future implementations of the tool should involve the MoH, NTD programme and other partners, in order for the outcomes to be owned by the MoH and therefore more likely to be acted upon to improve programme performance.

In conclusion, this study revealed that the majority of data reported in LF control programme in the study areas was inaccurate, and highlighted some programmatic challenges that must be addressed. At the time of this study, only five indicators could be assessed at a time using the DQA tool and perhaps provision could be made for many more indicators to be evaluated. The DQA tool holds tremendous value in evaluating NTD control programmes, and its use in indicator assessment points to its usefulness in assisting programme managers to address the issues of inaccurate reporting and data quality, following MDA. Using the tool is quite simple and it is recommended that sub-District, District, regional and national management levels use the tool in assessing their NTD programme performance. However, further sensitization and training on this tool for NTD programme personnel or teams at sub-national levels is recommended to ensure its use in the M&E of NTD control programmes. While the results from this study are informative, a more complete assessment of the LF Control Programme (involving the MoH, NTD programme and other NTD partners in the country) must be undertaken at all levels, in order to establish appropriate programmatic responses. All programme activities need to be closely supervised in order to ensure accurate data.
